# Hip instability and scoliosis in children with spinal muscular atrophy: a single center retrospective study in the United Arab Emirates

**DOI:** 10.3389/fped.2026.1732695

**Published:** 2026-04-20

**Authors:** Hagir Al-Dulaimi, Maryam Alabdullah, Salama AlGhanim, Haitham Elbashir, Ajay Dsouza, Ganga Rajapandian, Ibrar Majid, Sattar Alshryda, Mason Alnouri, Ali Nasreldien, Eurose Majadas, Noleen de Jager, Gail Alexander, Omer Al-Dulaimi, Abdulla AlGhanim, Zainab Al-Abdullah, Nidheesh Chencheri

**Affiliations:** 1 College of Medicine, Mohammed Bin Rashid University of Medicine and Health Sciences, Dubai Health, Dubai, United Arab Emirates; 2 Neurosciences, Al Jalila Children’s Specialty Hospital, Dubai Health, Dubai, United Arab Emirates; 3 Department of Radiology, Al Jalila Children’s Specialty Hospital, Dubai Health, Dubai, United Arab Emirates; 4 Department of Pediatric Orthopedics and Trauma, Al Jalila Children’s Specialty Hospital, Dubai Health, Dubai, United Arab Emirates; 5 Department of Rehabilitation, Dubai, United Arab Emirates; 6 Cardiff and Vale University Health Board, Cardiff, NHS Wales, United Kingdom; 7 College of Medicine and Health Sciences, United Arab Emirates University, Al Ain, United Arab Emirates

**Keywords:** disease modifying therapy, hip dislocation, hip subluxation, scoliosis, spinal muscular atrophy, survival motor neuron 2 gene

## Abstract

**Introduction:**

Hip instability is an important complication of spinal muscular atrophy (SMA), which leads to various functional impairments, including mobility challenges and difficulties with daily care. The primary objective of this study is to determine the prevalence and severity of hip instability in a cohort of SMA patients managed at a tertiary care center. The prevalence of scoliosis and its association with hip instability in children with SMA were also assessed.

**Methods:**

This is a retrospective cross-sectional study including children with a genetic diagnosis of SMA and available hip x-rays, conducted between 2018 and 2023. Patients were grouped based on SMA type and motor status (non-sitters, sitters, or walkers). Relevant parameters were collected including hip subluxation/dislocation, presence of scoliosis, number of *SMN2* copies and disease modifying therapies (DMTs) received.

**Results:**

Fifty-four children with types 1–3 SMA were included in this study. Hip dislocation/subluxation in both hips was most prevalent in type 1 SMA patients [Fisher's Exact Test (FET): *p* = 0.046]. There was no significant correlation between the presence of hip subluxation or dislocation and scoliosis, but patients with fewer *SMN2* copies were more likely to have scoliosis (*p* = 0.023). Several factors, including gender, age, motor status and presence of scoliosis were analysed, but none showed a statistically significant association with hip abnormalities.

**Discussion:**

To the best of our knowledge, this is the first study in the Middle East on hip instability and scoliosis in a cohort of children with SMA. Hip dislocation and/or subluxation is most common and most severe in type 1 SMA. The study's findings can aid the development of hip instability screening programs in SMA patients, enabling early intervention.

## Highlights

- Hip instability is most prevalent and severe in type 1 SMA patients.
- Prevalence of hip instability highlights the need for routine screening for SMA patients.
- An association between fewer *SMN2* copies and the presence of scoliosis.
- No significant correlation between DMTs and presence of hip instability.

## Introduction

Spinal muscular atrophy (SMA) is an autosomal recessive disorder characterized by progressive degeneration of alpha motor neurons in the spinal cord leading to muscle atrophy, weakness and paralysis (1). It is the leading genetic cause of death in children below the age of two years (2), with an estimated incidence of 1 in 10,000 live births (3).

The most common form of SMA results from a mutation or a deletion in both copies of the survival motor neuron 1 gene (*SMN1*) located on chromosome 5q. This leads to degeneration of the anterior horn cells of the spinal cord, which are largely responsible for skeletal muscle innervation. A second gene, *SMN2*, plays a role in the severity of the disease. *SMN2* is intact in all SMA patients but the number of copies can vary from zero to four copies on each chromosome, and SMA patients always carry at least one copy (4). *SMN2* encodes for a similar but a truncated version of the *SMN* protein therefore it provides limited function. As a result, a positive correlation between a higher *SMN2* copy number and milder phenotypes has been demonstrated (5).

Hip instability (subluxation and/or dislocation) is a significant manifestation of SMA. This problem arises from weakness of the key hip stabilization muscles, which leads to functional impairments including difficulties with perineal hygiene, seating, transfers, positioning, dressing and pain (6, 7). Hip instability that results from this weakness is classically quantified by the hip migration index, which is measured on an anterior-posterior (AP) radiograph (8).

Scoliosis is another major manifestation of the disease with a reported prevalence of 70%–100% of cases (7). Complications include functional deficits, pain and reduced lung volume (7). According to some studies, asymmetric weakness or unilateral hip dislocations can cause an initial curvature of the spine, an association that has been investigated in this study (7). Scoliosis is assessed by a whole spine x-ray, highlighting the importance of radiography in the assessment of SMA.

Due to the heterogeneous nature of the disease, SMA is categorized into five types based on the age of onset of their clinical presentation. Type 0 has a prenatal or neonatal onset and is the most severe but a very rare form of the disease. Type 1, which presents in the first six months of age, describes extremely weak infants unable to sit unsupported and is the most common form of SMA, accounting for around 58% of all cases. Type 2 is assigned to non-ambulant patients who are able to sit independently. Ambulant patients with childhood or adult onset are categorized as type 3 and type 4 respectively (9).

With the introduction of disease modifying therapies (DMT), namely Nusinersen (Spinraza), approved by the Food and Drug Association (FDA) in 2016 (10), Onasemnogene abeparvovec [Zolgensma, introduced at Al Jalila Children's Specialty Hospital in November 2020 (11)], and Risdiplam [Evrysdi, FDA approved in 2020 (12)] there have been significant improvements in life expectancy and quality of life, as well as a gain in motor skills (5). For example, type 2 patients are now acquiring the ability to walk, something which is characteristic of type 3 (13). Due to the deviation from the natural disease course, a newer classification for SMA has been introduced based on current motor status; non-sitters, sitters and walkers (13). The gain in ambulatory function brings new challenges such as different gaits observed in type 3 and treated type 2 (13). The effects of improvements of motor status on hip instability are not well understood, emphasising the importance of hip surveillance.

To our knowledge, there are no previous studies conducted in the Middle east evaluating hip instability in children with SMA. The aim of this study is to assess hip instability and scoliosis in children with Types 1, 2, and 3 SMA through radiological assessment.

## Study objectives

– **Primary Objective**:
  - To determine the prevalence and severity of hip instability in a cohort of SMA patients managed at a tertiary care center.
– **Secondary Objectives**:
  - To examine the relationship between SMA subtypes and the occurrence of hip instability.
  - To assess the prevalence of scoliosis and investigate its association with hip instability in children with SMA.
  - To investigate the association between SMN2 copies and radiographic indicators of disease severity.

## Materials and methods

### Study design and setting

This retrospective cross-sectional study analyzed data from children under the age of 18 years with a genetic diagnosis of 5q SMA. The patients were seen at the pediatric neurology and neurorehabilitation clinics of Al Jalila Children's Specialty Hospital in the United Arab Emirates between June 1st 2018, and June 1st 2023.

SMA patients are identified with having a homozygous deletion of SMA 1 gene mutation via Multiplex Ligation dependent Probe Amplification (MLPA)/qPCR testing method (14). All children with a genetic diagnosis of SMA who had a supine AP hip x-ray as a part of work-up were included in the study. These who did not have radiological examination of the hip were excluded from the study.

### Data collection

Data were extracted from the electronic medical database and transferred onto a Microsoft Excel sheet for data collection. SMA type (1, 2, 3) was determined by the age of onset of clinical presentation. Motor status (non-sitter, sitter, walker) of each patient was determined by a paediatric neurologist/neurorehabilitation consultant based on the patients' clinical profile.

Data on the use of DMTs (Nusinersen/Risdiplam) of each patient were also collected. Considering that other studies used a 6-month timeframe to demonstrate a noticeable response to DMTs, patients who had been taking DMTs for at least 6 months prior to the hip x-ray were classified as having used DMTs (15). In contrast, those who had either not taken DMTs or had been on them for less than 6 months before the hip x-ray were categorized as not having taken DMTs.

Patients' age and motor status were documented at the time the x-ray was performed. Radiographic measurements such as migration index (MI) and Cobb's angle of all available hips and spine x-rays with adequate projections of the pelvis and spine respectively were reported by a single paediatric radiology consultant to avoid inconsistency in the radiological reporting. x-rays were taken via the Philips x-ray machine as per hospital protocol. Migration index measures the percentage of the femoral head's containment within the acetabulum with respect to how lateral it is to Perkin's line on an AP view hip x-ray (7, 16). A migration index of 0%–33% indicates no subluxation, 33%–99% indicates subluxation and hip dislocation is diagnosed when the migration index is 100% or the femoral head is completely uncovered (7). Scoliosis was measured manually based on the Cobb criteria on AP spine x-rays. A Cobb's angle between 10 and 20 degrees is considered mild scoliosis, between 20 and 40 degrees is considered as moderate, and a Cobb's angle greater than 40 degrees is considered severe scoliosis (17).

### Statistical methods

All statistical analyses were performed using SPSS software version 28. Data was tested for normality using Shapiro–Wilk/Kolmogorov–Smirnov test as appropriate. Categorical variables such as degree of hip instability were tabulated as frequencies and percentages (%). Continuous variables such as migration index were presented as a mean ± standard deviation (SD). To determine statistically different means between three or more groups, ANOVA with pairwise comparisons test was used. Associations between categorical variables were analysed using the Chi-square test or Fisher's Exact Test (FET), with FET applied when any category contained fewer than 5 patients. Bivariate analysis and Pearson's Correlation Coefficient (r) was used to test for the significance and strength of the association between two continuous variables. Ordinal logistic regression analysis was done using STATA 16.0 software. A *p*-value of ≤0.05 was chosen to determine statistical significance. Missing data were excluded listwise.

### Sample size

The prevalence of hip instability has been reported to be 75% (18). Assuming a similar prevalence in our population, a sample size of approximately 72 subjects would be required to estimate this proportion with a precision of ±10%. If a precision of ±15% were considered as acceptable, the required sample size would be approximately 32 subjects. Based on these estimates, we elected to include 54 subjects, corresponding to an expected precision of approximately ±12%.

## Results

### Participants

**Figure 1:**
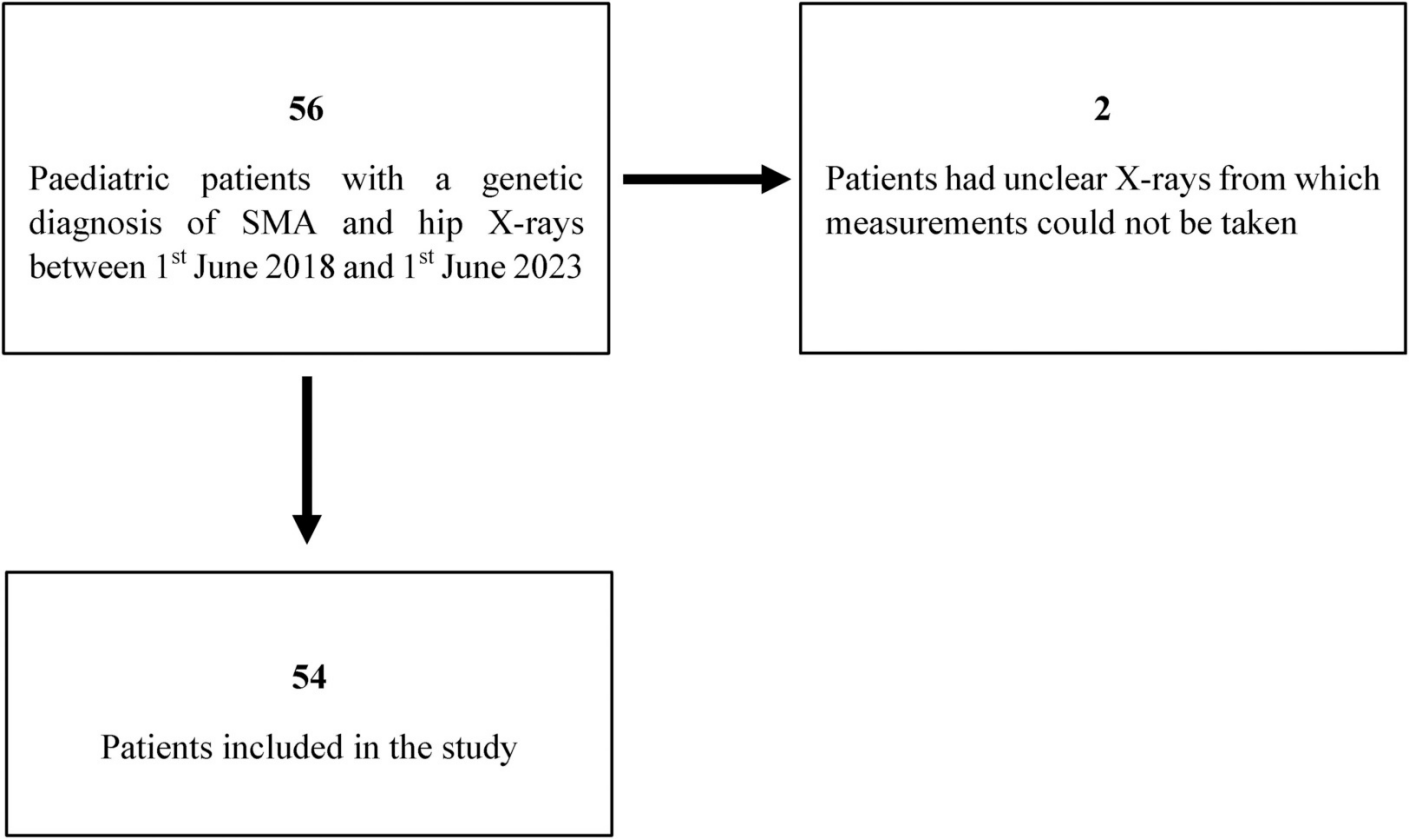
Flow chart of participants.

A total of 54 children with SMA types 1, 2 and 3 who had an x-ray of the hips were included in the study. Two patients had unclear x-rays due to inadequate projection of the hip joints which could not be accurately assessed, so were excluded from the study (Figure 1). The study included 25 males and 29 females. There were 34 individuals with type 1 SMA, 16 with type 2 and four with type 3. Patients were also categorized based on their motor status; there were 29 non-sitters, 20 sitters and five walkers. The mean (SD) age was 34 (27) months with a range from 3 to 180 months. Overall, eighteen (33%) patients had no subluxation, 16 had a unilateral subluxation, 17 had bilateral subluxation and three had bilateral hip dislocation. Spine x-rays were available for 41 patients, of whom 20 (50%) were diagnosed with scoliosis. Among these, 10 had mild scoliosis, seven had moderate scoliosis, and three had severe scoliosis.

Out of the 54 patients, 49 had received at least 1 dose of a DMT previously. Forty-one patients had received Nusinersen and 8 received Risdiplam. No patient received more than one type of DMT. Thirty-four patients had received DMTs for longer than 6 months, and these were considered as having taken DMTs. Of these, 33 had been treated with Nusinersen and one with Risdiplam. The age at initiation of DMT within this group ranged from 1 to 44 months, with a mean (SD) of 9.8 (9.8) months. The duration of therapy prior to radiographic assessment within this group ranged from 6 to 55 months, with a mean (SD) of 24.2 (11.5) months. With regards to the *SMN2* copies, 34 patients had 2 copies, 16 patients had 3 copies, and 4 had 4 copies (Table 1).

**Table 1:** Summary of patient data.

Characteristic		Frequency	Percentage
Gender	Male	25	46%
	Female	29	54%
SMA Type	Type 1	34	63%
	Type 2	16	30%
	Type 3	4	7%
Motor Status	Non-sitter	29	54%
	Sitter	20	37%
	Walker	5	9%
Age Category	0–23 months	22	41%
	24–47 months	23	42%
	48+ months	9	17%
Scoliosis	Yes	20	37%
	No	21	39%
	Unavailable Xray	13	24%
Hip Instability	No subluxation	18	33%
	Unilateral Subluxation	16	30%
	Bilateral Subluxation	17	31%
	Dislocation	3	6%
*SMN2* copies	2 copies	34	63%
	3 copies	16	30%
	4 copies	4	7%
DMT	Yes	34	63%
	No	20	37%

### Descriptive data

Hip instability, including subluxation and dislocation, were observed in 66.7% of our patients, with the highest prevalence in SMA type 2 (75%), followed by SMA type 1 (67.6%) and type 3 (25%). This difference in prevalence among the SMA types was not statistically significant (FET: *p* = 0.171). However, there was a significant correlation between the degree of hip instability and the type of SMA. Bilateral subluxation or dislocation was observed in 50% of SMA type 1 patients, compared to 19% in type 2% and 0% in type 3, indicating that SMA type 1 exhibited the highest degree of instability. This finding was statistically significant (FET: *p* = 0.046) (Table 2). Abnormal hips were observed in 61.8% of patients with two SMN2 copies, 75% of those with three copies, and 75% of those with four copies, with no statistically significant difference (FET: *p* = 0.729). A similar lack of significant association was observed with gender, age category, motor status and treatment status. Although 61% of treated patients had abnormal hips compared to 84.6% in the untreated group, this difference did not reach statistical significance (FET: *p* = 0.179). Variables were further analysed using ordinal logistic regression, with hip instability as the dependent variable and age, gender, motor status, SMA type, the number of *SMN2* copies, scoliosis and usage of DMTs as independent variables. Logistic regression showed that the children who presented with SMA type 1 are 28.5 (95% CI: 0.80, 1,017.8) times more likely to have hip instability as compared to children with SMA Type 3 (*p* = 0.066). As the SMN2 copies increased, the odds of hip instability increased by about 5 times (95% CI: 1.1, 22.1), which was statistically significant (*p* = 0.035) (Table 3).

**Table 2:** Association between type of SMA and degree of Hip instability.

Type of SMA	Hip Instability								Total
	No subluxation		Unilateral subluxation		Bilateral subluxation		Dislocation		
Type 1	11	(32%)	6	(18%)	14	(41%)	3	(9%)	34
Type 2	4	(25%)	9	(56%)	3	(19%)	0	(0%)	16
Type 3	3	(75%)	1	(25%)	0	(0%)	0	(0%)	4
Total	18		16		17		3		54

**Table 3:** Ordinal logistics regression analysis for Hip instability.

Risk variables	Odds ratio	95% CI		*P* value
		LL	UL	
Gender (Female)	2.82	0.71	11.13	0.14
SMA Type				
1	28.50	0.80	1,017.81	0.066
2	5.01	0.23	108.01	0.304
3	1.00			
SMN2 Copies	4.99	1.12	22.16	0.035
Motor Function Level	0.68	0.18	2.48	0.557
Age	1.40	0.59	3.32	0.441
Scoliosis	0.68	0.34	1.33	0.257
DMT (Did not take)	0.37	0.06	2.51	0.311

A lower number of *SMN2* copies was significantly associated with the presence of scoliosis (FET: *p* = 0.023). However, no significant association was found between the number of *SMN2* copies and hip instability. Among patients with scoliosis, 80% had only 2 copies of *SMN2*, 15% had 3 copies, and 5% had 4 copies (Table 4). Conversely, the likelihood of developing scoliosis also decreased with a higher number of *SMN2* copies: 66% of patients with 2 copies developed scoliosis, compared to 21.4% of those with 3 copies and 33.3% of those with 4 copies (FET: *p* = 0.019). No significant association was found between the presence of scoliosis and gender, age category, motor status, or treatment status, suggesting that these factors did not impact the prevalence of scoliosis. No association was found between scoliosis and hip subluxation or dislocation (FET: *p* = 1.0) (Table 5). However, spine radiographs were unavailable in 13 patients, which may limit interpretation of this finding.

**Table 4:** Association between SMN2 copies and presence of scoliosis.

Scoliosis	*SMN2* copies						Total
	2 Copies		3 Copies		4 Copies		
Yes	16	(80%)	3	(15%)	1	(5%)	20
No	8	(38%)	11	(52%)	2	(10%)	21
Total	24		14		3		41

**Table 5:** Association between scoliosis and degree of hip instability.

Scoliosis	Hip instability								Total
	No subluxation		Unilateral subluxation		Bilateral subluxation		Dislocation		
Yes	7	(50%)	6	(50%)	6	(50%)	1	(33.3%)	20
No	7	(50%)	6	(50%)	6	(50%)	2	(66.7%)	21
Total	14		12		12		3		41

## Discussion

Hip instability is an important complication of SMA, which leads to various functional impairments, including mobility challenges and difficulties with daily care (7). Advances in gene therapy have revolutionized SMA treatment by improving motor function, muscle strength, and quality of life (5). In the context of these therapeutic advances, our study aimed to assess hip instability and scoliosis in children with Types 1, 2, and 3 SMA.

SMA type 1 demonstrated the highest level of hip instability, with bilateral hip subluxation being the most common condition. Hip dislocation was noted only in type 1. This highlights the greater degree of instability in SMA type 1, a finding that was statistically significant. However, our study could not detect an association between DMTs and hip instability given the available data. Several factors, including gender, age category, motor status, presence of scoliosis, and treatment status, were analyzed, but none showed a statistically significant association with hip abnormalities. Interestingly, motor status and treatment status demonstrated expected trends: walkers and those receiving treatment were less likely to have hip abnormalities, though these trends did not reach statistical significance.

A recent study by Kuong and colleagues found that the initiation of Nusinersen after the onset of motor weakness showed no statistically significant improvement in hip instability radiologically among type 2 patients (19). Similarly, our study did not observe a notable association between DMT use and hip instability, although the majority of our patient population was type 1 SMA. A Cochrane systematic review concluded moderate evidence on the benefit of DMTs improving hip instability in types 1,2 and 3 (20, 21). Therefore, the true influence of DMT on hip instability is yet to be established.

A study conducted by Krajewski and colleagues portrayed a significant correlation between increasing age and a higher migration index for types 1, 2 and 3 (22). Granata and colleagues reported similar findings of a linear correlation between migration index and age in sitters (23). However, our study found both of these findings to be insignificant although the majority of our patient cohort was less than 4 years old. Moreover, our study included patients who began DMTs at various ages, which can change the natural history of their disease (24).

In this study, only 49% of patients with available spine Xrays had scoliosis, which contrasts with findings from previous studies (7, 25). In a study by Driscoll et al., scoliosis was prevalent in 70%–100% of SMA patients (7). This difference could be attributed to the fact that these epidemiological data were reported before the introduction of DMTs, such as Nusinersen (10), which could have altered the disease's natural progression. In another more recent study, scoliosis was reported in 79% of SMA patients of types 1, 2 and 3 (25). This is higher than the prevalence in our study, which may be due to the fact that the age of onset of scoliosis was reported at 7.9 years while the mean age of patients in our study was 2.8 years. Therefore, it is likely that many of our patients have not yet developed scoliosis. This younger cohort of patients may be due to increased awareness and earlier detection, which facilitated timely referral to a tertiary care center offering DMTs.

Children with a lower number of *SMN2* copies showed significant association with the presence of scoliosis, but not with hip instability. Among patients with scoliosis, 80% had only two copies of *SMN2*, 15% had three copies, and 5% had four copies This relationship could be explained by the fact that more severe phenotypes are expected in those with fewer *SMN2* copies (5). Therefore, truncal muscle weakness is more profound, leading to scoliosis. These results are clinically relevant as early surgical correction of scoliosis has been found to have better outcomes (4), including sitting comfort and quality of life.

A study by Driscoll et al. suggested that unilateral hip instability can cause an initial curve in the spine (7). Another study identified hip displacement as a risk factor for scoliosis (25). However, we found no correlation between the presence of hip instability and scoliosis. Further longitudinal studies may be needed to confirm whether an initial unilateral subluxation/dislocation plays a role in the development of scoliosis.

## Clinical implications

Hip instability is one of the most common manifestations of SMA, yet hip screening is currently not a standardized procedure for children with SMA. Strategies to improve hip stability need to be implemented to prevent the development of hip abnormalities. This study highlights the importance of implementing hip screening procedures, particularly in type 1 SMA, given its increased severity. Early identification of hip subluxation facilitates earlier intervention leading to better patient outcomes.

The introduction of DMTs appears to have altered the natural course of SMA, as seen in the lower prevalence of scoliosis compared to historical data. However, this may also reflect a need to adapt clinical expectations and treatment protocols to the evolving phenotype of SMA in the post-DMT era. *SMN2* copy number could serve as a valuable predictive marker for scoliosis risk, aiding in the early identification of at-risk patients and guiding timely interventions and monitoring strategies.

Our study found no significant association between DMT use and improved hip stability or reduced prevalence of scoliosis. However, given the study design, these findings do not allow conclusions about the effectiveness of DMTs on musculoskeletal outcomes. Further prospective studies are needed to better clarify the relationship between DMT use and orthopaedic complications.

## Strengths, limitations and generalisabiliy

This study is unique in that it included children who received recently developed DMTs, specifically Risdiplam and Nusinersen. In addition, the sample size was sufficient given the rarity of SMA. However, despite the strengths of the study, there are some limitations. The study included a heterogeneous sample, which had some uncontrolled confounding factors (the children received different numbers of DMT doses and at different times), and this made it challenging to measure the outcomes and risk factors. Therefore, the findings were presented as potential associations warranting further investigation, rather than establishing causal relationships between variables. As this is a retrospective study, we were limited by the availability and completeness of historical medical records and imaging. Pre-treatment baseline x-rays were not consistently available, which affected our ability to assess the progression of scoliosis in relation to treatment initiation. It was difficult to assess the effects of DMTs on hip instability in the sub-group of patients who have received treatment, due to the fact that the majority of these patients did not have pre-treatment x-Rays for comparison.

## Areas for future research

With the recent U.S. Food and Drug Administration approval of DMTs, understanding the effects that these genetic therapies will have on hip instability and scoliosis is challenging (26); therefore, it is important that future studies evaluate the outcomes in children who have received the DMTs and compare the results with previous radiological data. Future studies may want to consider conducting longitudinal studies, in order to obtain serial follow-up x-rays for analyzing the effects of the DMTs on hip instability in these patients.

## Conclusion

This study has, to our knowledge, provided data on hip instability and scoliosis in children with SMA using proper radiographic protocols for the first time in the Middle East. Maintaining hip stability is important considering improved life expectancy and increased probability of ambulation after the introduction of DMTs. Hip instability seems to be most severe in type 1 SMA patients, with increased presence of bilateral subluxation or dislocation compared to other SMA types. This study sheds light on the importance of screening for hip instabilities in children with SMA for timely intervention.

## Data Availability

The raw data supporting the conclusions of this article will be made available by the authors, without undue reservation.
