# Supplementary material for: Hip instability and scoliosis in children with spinal muscular atrophy: a single center retrospective study in the United Arab Emirates
Source: Front Pediatr. 2026 Apr 20;14:1732695. doi: 10.3389/fped.2026.1732695 (PMC13136262; doi:10.3389/fped.2026.1732695)
Supplement: Supplementary file 1 [file Table1.docx]

**Supplementary Table 1:** Summary of DMT profiles

| **Patient** | **Age (months)** | **Age at initiation of DMT (months)** | **DMT received** | **Duration on DMT (months)** | **Doses of Nusinersen** | **DMT longer than 6 months** |
| --- | --- | --- | --- | --- | --- | --- |
| 1 | 3 | NA | NA | NA | NA | No |
| 2 | 12 | 8 | Nusinersen | 4 | 4 | No |
| 3 | 20 | 18 | Nusinersen | 2 | 4 | No |
| 4 | 22 | 4 | Nusinersen | 18 | 6 | Yes |
| 5 | 24 | 15 | Nusinersen | 9 | 5 | Yes |
| 6 | 36 | NA | NA | NA | NA | No |
| 7 | 10 | 5 | Nusinersen | 5 | 3 | No |
| 8 | 19 | 17 | Risdiplam | 2 | NA | No |
| 9 | 24 | NA | NA | NA | NA | No |
| 10 | 36 | 9 | Nusinersen | 27 | 7 | Yes |
| 11 | 37 | 18 | Nusinersen | 19 | 6 | Yes |
| 12 | 30 | 4 | Nusinersen | 26 | 9 | Yes |
| 13 | 9 | 1 | Nusinersen | 8 | 5 | Yes |
| 14 | 9 | 6 | Risdiplam | 3 | NA | No |
| 15 | 39 | 3 | Nusinersen | 36 | 12 | Yes |
| 16 | 29 | 7 | Nusinersen | 22 | 7 | Yes |
| 17 | 12 | 8 | Risdiplam | 4 | NA | No |
| 18 | 38 | 18 | Nusinersen | 20 | 7 | Yes |
| 19 | 34 | 7 | Nusinersen | 27 | 9 | Yes |
| 20 | 33 | 4 | Nusinersen | 29 | 9 | Yes |
| 21 | 12 | NA | NA | NA | NA | No |
| 22 | 20 | 3 | Nusinersen | 17 | 6 | Yes |
| 23 | 27 | 3 | Nusinersen | 24 | 6 | Yes |
| 24 | 42 | 19 | Nusinersen | 23 | 7 | Yes |
| 25 | 31 | 3 | Nusinersen | 28 | 10 | Yes |
| 26 | 35 | 3 | Nusinersen | 32 | 10 | Yes |
| 27 | 22 | 14 | Nusinersen | 8 | 5 | Yes |
| 28 | 33 | 4 | Nusinersen | 29 | 8 | Yes |
| 29 | 26 | 16 | Nusinersen | 10 | 5 | Yes |
| 30 | 49 | 8 | Nusinersen | 41 | 11 | Yes |
| 31 | 22 | 22 | Risdiplam | 0 | NA | No |
| 32 | 22 | 22 | Risdiplam | 0 | NA | No |
| 33 | 14 | 4 | Nusinersen | 10 | 7 | Yes |
| 34 | 16 | 14 | Nusinersen | 2 | 4 | No |
| 35 | 37 | 4 | Nusinersen | 33 | 10 | Yes |
| 36 | 20 | 18 | Risdiplam | 2 | NA | No |
| 37 | 58 | 3 | Nusinersen | 55 | 17 | Yes |
| 38 | 41 | 12 | Nusinersen | 29 | 7 | Yes |
| 39 | 36 | 8 | Nusinersen | 28 | 7 | Yes |
| 40 | 62 | 44 | Nusinersen | 18 | 11 | Yes |
| 41 | 63 | 36 | Nusinersen | 27 | 6 | Yes |
| 42 | 32 | 2 | Nusinersen | 30 | 10 | Yes |
| 43 | 46 | 18 | Nusinersen | 28 | 7 | Yes |
| 44 | 16 | 3 | Nusinersen | 13 | 6 | Yes |
| 45 | 10 | 10 | Risdiplam | 0 | NA | No |
| 46 | 10 | 1 | Nusinersen | 9 | 5 | Yes |
| 47 | 23 | NA | NA | NA | NA | No |
| 48 | 39 | 18 | Risdiplam | 21 | NA | Yes |
| 49 | 15 | 1 | Nusinersen | 14 | 7 | Yes |
| 50 | 78 | 73 | Nusinersen | 5 | NA* | No |
| 51 | 78 | 73 | Nusinersen | 5 | NA* | No |
| 52 | 78 | 74 | Nusinersen | 4 | NA* | No |
| 53 | 180 | 175 | Nusinersen | 5 | NA* | No |
| 54 | 71 | 17 | Nusinersen | 54 | 11 | Yes |

* No record of doses of Nusinersen received for these patients
